# Splicing-dependent expression of microRNAs of mirtron origin in human digestive and excretory system cancer cells

**DOI:** 10.1186/s13148-016-0200-y

**Published:** 2016-03-25

**Authors:** Stasė Butkytė, Laurynas Čiupas, Eglė Jakubauskienė, Laurynas Vilys, Paulius Mocevicius, Arvydas Kanopka, Giedrius Vilkaitis

**Affiliations:** Department of Biological DNA Modification, Institute of Biotechnology, Vilnius University, Vilnius, Lithuania; Department of Immunology and Cell Biology, Institute of Biotechnology, Vilnius University, Vilnius, Lithuania; Institute for Digestive Research, Lithuanian University of Health Sciences, Kaunas, Lithuania

**Keywords:** Mirtron, microRNA, miRNA, Post-transcriptional RNA processing, Splicing factor, Human cancer

## Abstract

**Background:**

An abundant class of intronic microRNAs (miRNAs) undergoes atypical Drosha-independent biogenesis in which the spliceosome governs the excision of hairpin miRNA precursors, called mirtrons. Although nearly 500 splicing-dependent miRNA candidates have been recently predicted via bioinformatic analysis of human RNA-Seq datasets, only a few of them have been experimentally validated. The detailed mechanism of miRNA processing by the splicing machinery and the roles of mirtronic miRNAs in cancer are yet to be uncovered.

**Methods:**

We experimentally examined whether biogenesis of certain miRNAs is under a splicing control by analyzing their expression levels in response to alterations in the 5′- and 3′-splice sites of a series of intron-containing minigenes carrying appropriate miRNAs. The expression levels of the miRNAs processed from mirtrons were determined by quantitative real-time PCR in five digestive tract (pancreas PANC-1, SU.86.86, T3M4, stomach KATOIII, colon HCT116) and two excretory system (kidney CaKi-1, 786-O) carcinoma cell lines as well as in pancreatic, stomach, and colorectal tumors. Transiently expressed SRSF1 and SRSF2 splicing factors were quantified by western blotting in the nuclear fractions of HCT116 cells.

**Results:**

We found that biogenesis of the human hsa-miR-1227-3p, hsa-miR-1229-3p, and hsa-miR-1236-3p is splicing-dependent; therefore, these miRNAs can be assigned to the class of miRNAs processed by a non-canonical mirtron pathway. The expression analysis revealed a differential regulation of human mirtronic miRNAs in various cancer cell lines and tumors. In particular, hsa-miR-1229-3p is selectively upregulated in the pancreatic and stomach cancer cell lines derived from metastatic sites. Compared with the healthy controls, the expression of hsa-miR-1226-3p was significantly higher in stomach tumors but extensively downregulated in colorectal tumors. Furthermore, we provided evidence that overexpression of SRSF1 or SRSF2 can upregulate the processing of individual mirtronic miRNAs in HCT116 cells.

**Conclusions:**

An interplay of different splicing factors, such as SRSF1 or SRSF2, may alter the levels of miRNAs of mirtron origin in a cell. Our findings underline the specific expression profiles of mirtronic miRNAs in colorectal, stomach, and pancreatic cancer.

**Electronic supplementary material:**

The online version of this article (doi:10.1186/s13148-016-0200-y) contains supplementary material, which is available to authorized users.

## Background

All multi-exon human genes undergo constitutive and alternative splicing, a very precise process which is crucial for regulation of gene expression and generation of proteomic and functional diversity [1]. In the cell, RNA splicing takes place in the nucleus within a large macromolecular complex, the spliceosome, which consists of five small nuclear ribonucleoproteins (snRNPs) and over a few hundred auxiliary proteins [2]. Somatic mutations or dramatic alterations in the amount of splicing factors under pathologic conditions, particularly in human cancers, have been observed, thus resulting in abnormal expression of tumor suppressors or oncogenes [3–5]. Furthermore, various types of cancer have been linked to dysregulation of microRNAs (miRNAs), short non-coding RNAs that post-transcriptionally regulate gene expression in mammals [6]. Although the majority of miRNA genes possess their own internal promoters that are regulated by different transcription and epigenetic factors, approximately one third of them are processed from introns of protein-coding or long non-coding RNAs genes [7]. That provides means for coupled regulation of miRNA maturation and mRNA splicing. However, the spatiotemporal control of miRNA expression by splicing factors has not been elucidated.

Canonical pri-miRNA is cleaved by Drosha/DGCR8 complex to form pre-miRNA hairpin, which is recognized by Exportin-5 and transferred to the cytoplasm. After processing by Dicer ribonuclease, the guide miRNA strand of mature miRNA/miRNA* duplex is incorporated into a RNA-induced silencing complex (RISC). An alternative pathway of miRNA biogenesis was lately described in eukaryotes [8, 9]. Short introns containing miRNAs, termed mirtrons, are spliced and debranched into pre-miRNA hairpin mimics that appear to bypass Drosha cleavage (Additional file 1: Figure S1). Debranched mirtrons enter the canonical miRNA pathway during nuclear export. Mirtrons were originally described in flies and worms [10, 11], but similar loci (i.e., short hairpin introns associated with small RNA reads extending to intronic termini) were later predicted in a range of plants and mammalians, including humans [12–14]. Based on the nature of terminal overhangs, four distinct mirtron subtypes are recognized: conventional (hairpin without tails), 3′-tailed, 5′-tailed, and two-tailed [14].

Despite the fact that the first splicing-dependent human mirtrons were annotated in 2007 [15] and a large number of splicing-derived miRNAs have been predicted by bioinformatic analysis of deep sequencing data [12, 14], only two of them, hsa-miR-877-5p and hsa-miR-1226-3p, were experimentally proven to be directly affected by splicing [16, 17]. 478 mirtron candidates predicted by Wen et al. [14] could cover a significant part of the total human miRNA population (2588 mature miRNA entries according to miRBase release 21 [18]), but little is still known about their impact on the expression of disease-related genes. It is currently shown that the mirtronic miRNA hsa-miR-1226-3p may function as an oncosuppressor by downregulating the mucin 1 oncoprotein and the hsa-miR-1226-3p level is decreased in breast cancer cells [19]. hsa-miR-1236-3p processed from a putative mirtron downregulates alpha fetoprotein, thus leading to the inhibition of the PI3K/Akt pathway in hepatoma cancer cell lines [20], and negatively regulates the vascular endothelial growth factor receptor VEGFR-3 during inflammatory lymphangiogenesis [21]. In addition, a miR-1236-3p antisense oligonucleotide inhibits the glioma tumor cell growth and proliferation [22]. Overexpression of hsa-miR-877-5p was observed in metastatic melanoma [23] and endometrial serous adenocarcinomas [24], but no biological function was determined. Yet, very little is known about the expression profiles of mirtron-derived miRNAs in tumors or cancer cells*.* There is a high possibility that the expression levels of splicing factors can not only affect alternative pre-mRNA splicing but cause changes in mirtronic miRNA expression as well.

In this study, we examined eight putative mirtrons and provided experimental evidence that human hsa-miR-1227-3p, hsa-miR-1229-3p, and hsa-miR-1236-3p could be assigned to the class of mirtronic miRNAs. Digestive and excretory system cancer cell lines, as well as digestive system tumors tissues, display varying expression profiles of the previously identified hsa-miR-1226-3p and the two newly validated mirtronic miRNAs. Finally, we found that overexpression of well-known splicing factors SRSF1 and SRSF2 increased the abundance of some mirtron-derived miRNAs in colorectal HCT116 cancer cells.

## Results

### Experimental validation of new splicing-dependent human mirtronic miRNAs

**Fig. 1:**
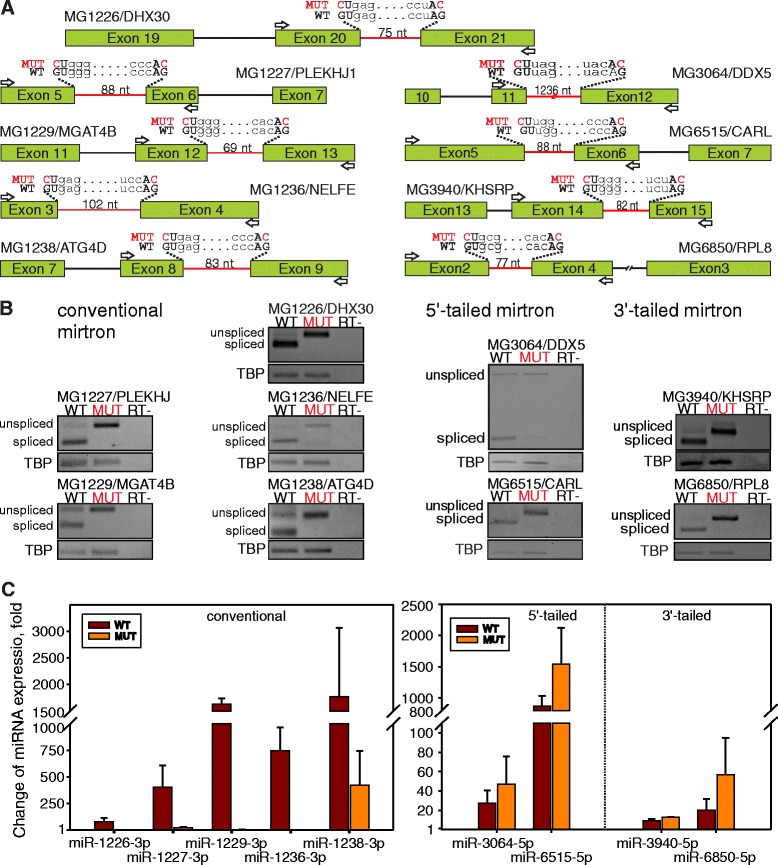
Identification of splicing-dependent miRNAs processed from mirtrons. **a** Schematic representation of exon–intron structures of analyzed human minigenes. *Boxes* and *lines* indicate exons and introns, respectively. Introns containing validated mirtronic miRNA hsa-mir-1226-3p or predicted miRNAs hsa-mir-1227-3p, hsa-mir-1229-3p, hsa-mir-1236-3p, and hsa-mir-1238-3p processed from conventional; hsa-miR-3064-5p and hsa-miR-6515-5p from 5′-tailed; and hsa-miR-3940-5p and hsa-miR-6850-5p from 3′-tailed mirtrons are depicted as *red lines*. WT, wild-type sequences, and MUT, minigenes carrying the double mutant with GT to CT and AG to AC changes (affected nucleotides are marked in *red*), in 5′-donor and 3′-acceptor splice sites, respectively. **b** Splicing analysis of minigene transcripts in transfected HCT116 cells. The position of oligonucleotides used for RT-PCR analysis of mRNA transcripts produced by minigene constructs is indicated by *white arrows* in section a. Control reactions, *RT*, were performed without prior reverse transcription to exclude DNA contamination. Unspliced form indicates transcript retaining mirtronic intron; spliced, intron is excised. *TBP *— loading control. **c** miRNA expression in colorectal cancer HCT116 cell line transfected with native (WT) or splicing-deficient (MUT) minigenes was analyzed using reverse transcription-quantitative polymerase chain reaction (RT-qPCR). Expression level of RNU48 was used as endogenous reference for data normalization. The experiments were performed in at least three biological replicates. The *error bars* represent calculated values for standard deviation

The vast majority of nearly 500 mirtron-derived miRNA candidates, except hsa-miR-887 and hsa-miR-1226-3p processed from conventional mirtrons, are still listed as experimentally unverified [14, 16, 17]. In order to expand the number of comprehensively validated miRNAs of mirtron origin, we examined eight putative human mirtrons ascribed to three different subtypes (Additional file 1: Figure S2). Putative human conventional mirtron-derived hsa-miR-1227-3p, hsa-miR-1229-3p, hsa-miR-1236-3p, and hsa-miR-1238-3p [15] and 3′-tailed mirtron-derived hsa-miR-3940-5p and hsa-miR-6850-5p were identified in short 69–102 nucleotide introns, whereas hsa-miR-3064-5p and hsa-miR-6515-5p were processed from both long (1236 nt) and short (88 nt) 5′-tailed mirtrons [12]. To establish dependence of their biogenesis on mRNA splicing, we constructed plasmids harboring minigenes of two or one intron spanned by three and two coding exons, respectively (Fig. 1a). Wild-type (WT) minigenes encompassed the natural introns while MUT variants of minigenes contained the intron, which hosted miRNA, with mutations affecting the G residues at 5′ splice donor (GU changed to CU) and 3′ splice acceptor (AG changed to AC) sites. A plasmid with the MG1226/DHX30 minigene containing the functionally proven mirtronic hsa-miR-1226-3p [16] was used as a positive control. As shown in Fig. 1b, the introns are effectively excised in the majority of the analyzed mRNAs processed from plasmids with WT minigenes in colorectal carcinoma HCT116 cells. In contrast, mRNAs from the MUT variants retained the unspliced exon–intron–exon structure. No reverse transcription (RT)-PCR products were detected in the control samples obtained from cells transfected with an insert-less vector (data not shown) under similar reaction conditions, confirming that the majority of target mRNAs in cells were synthesized from the analyzed minigenes.

Simultaneous quantitation of individual miRNAs by real-time qPCR analysis revealed a 10–2000-fold enrichment of all tested intronic miRNAs when a plasmid with the WT minigene structure was transfected into the HCT116 cell line (Table 1 and Fig. 1c, brown bars). These results provide convincing evidence that the tested intronic regions generate specific miRNAs. A different outcome was observed for miRNA formation in cells transfected with the splicing-deficient MUT minigenes (Fig. 1c, orange bars). The expression levels of hsa-miR-1226-3p, hsa-miR-1227-3p, hsa-miR-1229-3p, and hsa-miR-1236-3p dramatically decreased and comprised less than 5 % of the levels observed for the WT minigenes (Table 1). These results convincingly confirmed that the biogenesis of hsa-miR-1226-3p, hsa-miR-1227-3p, hsa-miR-1229-3p, and hsa-miR-1236-3p is strictly splicing-dependent, thus the efficiency of the splicing machinery defines the quantity of these miRNAs. The hsa-miR-1238-3p expression level in the cell lines containing the intron excision-deficient MUT variant of minigene was more than 400-fold higher as compared to that in cells lacking the minigene. Different results were obtained with 5′- and 3′-tailed mirtrons, where disruption of the splicing sites did not inhibit the accumulation of hsa-miR-3064-5p, hsa-miR-6515-5p, hsa-miR-3940-5p, and hsa-miR-6850-5p (Fig. 1). Due to the observed splicing-independent maturation, these miRNAs cannot be considered *bona fide* mirtronic miRNAs.

**Table 1:** Alterations of miRNA expression level in cells transfected with native (WT) or splicing-deficient (MUT) minigenes

MicroRNA	WT minigene line^a^	MUT minigene line^a^	MUT/WT, %
Hosted on conventional mirtron			
hsa-miR-1226-3p	81 ± 36	1.5 ± 0.3	1.9
hsa-miR-1227-3p	405 ± 204	24 ± 8	5.9
hsa-miR-1229-3p	1633 ± 106	4.1 ± 2.1	0.3
hsa-miR-1236-3p	747 ± 217	2.6 ± 0.5	0.3
hsa-miR-1238-3p	1772 ± 1289	425 ± 322	24
Hosted on 5′-tailed mirtron			
hsa-miR-3064-5p	27 ± 13	47 ± 29	173
hsa-miR-6515-5p	875 ± 162	1541 ± 576	176
Hosted on 3′-tailed mirtron			
hsa-miR-3940-5p	9.1 ± 1.7	13 ± 0.3	141
hsa-miR-6850-3p	20 ± 12	57 ± 38	286

^a^Fold change

### Differential expression of miRNAs processed from conventional mirtrons in cancer cell lines

**Fig. 2:**
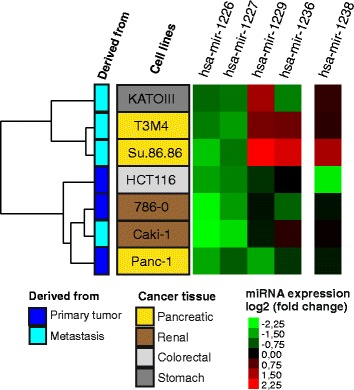
Heatmap of mirtronic miRNA expression in human cancer cell lines. Cell lines are in *rows*, miRNAs in *columns*. The relative expression of each miRNA was determined by real-time PCR; data are presented as log_2_ fold changes relative to embryonic cell line HEK293A. The *heatmap colors* represent relative miRNA expression as indicated in the color key: *red*, higher expression; *green*, lower expression; and *black*, no difference. Dendrogram of hierarchy clustering analysis is presented at the left

While a role of canonical Drosha/DGCR8-dependent miRNAs in human diseases is well recognized, there is much to be learned about the implications of mirtronic miRNAs in cancer development and their possible diagnostic potential. Carcinoma cell lines derived from various digestive system organs (pancreas PANC-1, SU.86.86, T3M4, stomach KATOIII, colon HCT116) or the excretory system (kidney CaKi-1, 786-O) were chosen to define whether the expression of the human mirtronic miRNAs hsa-miR-1226-3p, hsa-miR-1227-3p, hsa-miR-1229-3p, and hsa-miR-1236-3p and canonical hsa-miR-1238-3p varies between the cancerous cells. The human embryonic kidney HEK 293A cells were used as a reference cell line to which the other cell lines were compared. The results are summarized in Fig. 2, Table 2, and Additional file 1: Figure S3.

**Table 2:** Differential expression of mirtronic miRNAs in pancreas, kidney, colon, and stomach cancer cell lines

Organ	Cell line	miRNAs of mirtron origin				Non-mirtronic miRNA
		miR-1226-3p	miR-1227-3p	miR-1229-3p	miR-1236-3p	miR-1238-3p
Pancreas	PANC-1	−2.8 ± 1.8^**a**^	−1.7 ± 0.3^**a**^	−2.8 ± 0.9^**a**^	−1.4 ± 0.2	−1.2 ± 0.6
	SU.86.86	−3.4 ± 0.9^**a**^	−2.1 ± 0.4^**a**^	+5.0 ± 2.7^**b**^	+3.8 ± 0.6^**b**^	+2.8 ± 0.9^**b**^
	T3M4	−2.2 ± 0.4^**a**^	−2.6 ± 0.4^**a**^	+2.1 ± 0.3^**b**^	+1.9 ± 0.4^**b**^	+1.3 ± 0.2
Kidney	CaKi-1	−4.9 ± 2.1^**a**^	−3.9 ± 1.6^**a**^	−1.2 ± 0.6	+1.3 ± 0.7	1.0 ± 0.8
	786-O	−4.6 ± 2.2^**a**^	−2.6 ± 0.7^**a**^	−1.1 ± 0.2	−1.8 ± 0.2^**a**^	−1.1 ± 0.2
Colon	HCT116	−2.7 ± 0.8^**a**^	−2.2 ± 0.8^**a**^	−1.3 ± 0.2	1.0 ± 0.7	−4.2 ± 0.3^**a**^
Stomach	KATOIII	−1.9 ± 0.3^**a**^	−2.0 ± 0.4^**a**^	+2.7 ± 0.3^**b**^	−2.2 ± 0.3^**a**^	+1.3 ± 0.0

Fold changes higher than 1.5 were statistically significant (*p* < 0.05)

^**a**^Reduced expression compared to the non-cancerous HEK 293A cells

^**b**^Enhanced expression

The expression pattern of the tested mirtron-derived miRNAs revealed a unique miRNA signature of individual cancer cell lines (Fig. 2). The abundance of two of them, hsa-miR-1226-3p and hsa-miR-1227-3p, was reduced two- to five-fold in all cancer cell lines relative to the embryonic kidney HEK 293A cells. The highest downregulation was observed in the kidney carcinoma CaKi-1 and 786-O cells. In contrast, hsa-miR-1229-3p and hsa-miR-1236-3p showed significant variations among cancer cell lines and have a potential to be exploited for profiling specific cancer cell types. Correlations were found between the hsa-miR-1229-3p expression pattern and tissue origin — a steep increase in the mirtronic miRNA abundance was detected in three lines (SU.86.86, T3M4, and KATOIII) derived from metastatic sites as opposed to those derived from primary tumors. In particular, the PANC-1 cells (established from pancreatic carcinoma of ductal origin) showed a 14-fold reduction of this mirtronic miRNA as compared to the SU.86.86 cells (derived from pancreatic ductal carcinoma metastatic site in the liver). Moreover, hierarchical clustering based on relative expression variations for mirtronic miRNAs identified the majority of the cell lines derived from metastatic sites as a unique group (Fig. 2). However, further studies are required to ascertain whether hsa-miR-1229-3p is selectively upregulated in metastatic tumor tissues.

The levels of canonical hsa-miR-1238-3p expression in the majority of cancer cells, with the exception of SU.86.86 and HCT116, were similar to embryonic HEK 293A cells.

### Expression profiling of mirtronic miRNAs in pancreatic, colorectal, and stomach tumors

To further explore the impact of mirtronic miRNAs on human cancer development, we analyzed whether mirtron-derived miRNAs are differentially expressed in digestive system tumors (pancreatic, colorectal, and stomach) as compared to normal solid tissue samples of the same organ.

**Fig. 3:**
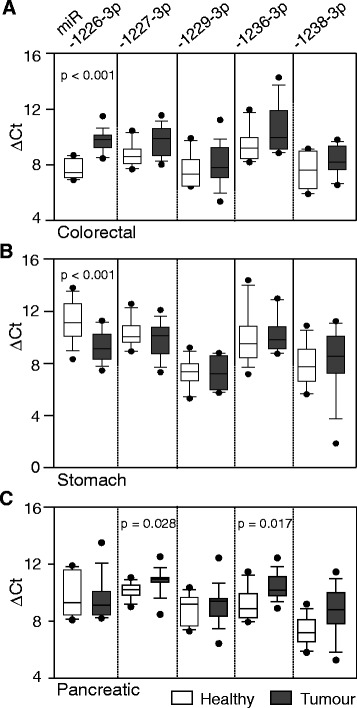
Variability in expression of mirtronic miRNAs in colorectal (**a**), stomach (**b**), and pancreatic adenocarcinoma (**c**) tissues. *∆C*_t_ values are quantified relative to RNU48 (*∆C*_t_ = *∆C*_miR_ − *∆C*_RNU48_). The *line* within a box marks the median value, the *boundaries* of the box indicate the 25th and 75th percentiles, the *asterisks* indicate the 90th and 10th percentiles, and the *circles* represent outliers. *p* values on top of the bars mark statistically significant differences between pairs. *White vertical boxplots* represent data from healthy tissues; *gray* from tumors

The tissue analysis results revealed that tumors of different organs have a characteristic signature of mirtronic miRNA expression (Fig. 3 and Table 3). We found that only one out of five tested miRNAs, hsa-miR-1226-3p, was significantly under-expressed in the colorectal tumor (*p* < 0.001). Conversely, the expression level of this miRNA was profoundly elevated in stomach cancer compared to that in non-tumor tissues (*p* < 0.001). Whereas steady levels of hsa-miR-1226-3p were detected in pancreatic cancer and healthy pancreatic tissues, two other mirtronic miRNAs, hsa-miR-1227-3p (*p* = 0.028) and hsa-miR-1236-3p (*p* = 0.017), were significantly downregulated in this tumor type. Thus, different expression profiles of mirtronic miRNAs were observed not only in cancerous cell lines but also in digestive organ tumors.

**Table 3:** Expression of mirtronic miRNAs in colorectal, stomach, and pancreatic cancerous tissues compared to healthy tissues

Cancerous tissue	miRNAs of mirtron origin				Non-mirtronic miRNA
	miR-1226-3p	miR-1227-3p	miR-1229-3p	miR-1236-3p	miR-1238-3p
Colorectal	**−**	No change	No change	No change	No change
Stomach	**+**	No change	No change	No change	No change
Pancreatic	No change	**−**	No change	**−**	No change

Data were considered significant for *p* < 0.05

− reduced expression compared to the non-cancerous tissue, + increased expression

### Effect of splicing factors SRSF1 and SRSF2 on biogenesis of mirtronic miRNAs

Despite a considerable progress in the study of biogenesis of mirtron-derived miRNAs, the factors responsible for mirtronic intron splicing are not known. Commonly, the efficiency of the macromolecular spliceosome complex is controlled by associated protein cofactors called splicing factors (SFs). However, it is not known whether the splicing proteins directly affect the biogenesis of mirtronic miRNAs in human cells. To evaluate the regulatory function of specific SF in maturation of mirtron-derived miRNAs, we overexpressed two essential sequence-specific SFs of serine-arginine-rich (SR) protein family, SRSF1 (also known as SF2/ASF) and the SRSF2 (also known as SC35), in human HCT116 cells and measured cellular levels of three splicing-dependent miRNAs, hsa-miR-1226-3p, hsa-miR-1227-3p, and hsa-miR-1229-3p. These prototypical SR proteins play an important role in the regulation of both constitutive and alternative pre-mRNA splicing [25]. In addition, SRSF1 is also involved in pri-miRNA processing [26].

**Fig. 4:**
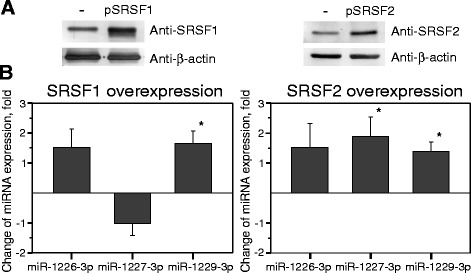
Different splicing factors can be involved in the biogenesis of specific mirtronic miRNAs. **a** The amount of SRSF1 and SRSF2 proteins markedly increased in transfected HCT116 cell line. Western-blot analysis was carried out on nuclear extracts of cells transfected with empty vector pcDNA3 (*-*) or plasmid containing recombinant SRSF1 gene (pSRSF1) or SRSF2 gene (pSRSF2). Beta-actin was used as an internal loading control. **b** Splicing factor-dependent alterations of endogenous mirtronic miRNA expression levels were determined using RT-qPCR. The mean values were calculated for three (*SRSF1*) and five (*SRSF2*) biological replicates. *Statistically significant results, *p* < 0.05

Western-blot analysis revealed a 3.2- and 1.6-fold increase in SRSF1 and SRSF2 protein yield in the nuclear extracts, respectively, after transfection of human HCT116 cells with the pSRSF1 or pSRSF2 constructs (Fig. 4a). Since the RNA splicing occurs within the nucleus, it is plausible that only biologically active intranuclear fractions of cellular SF were quantified in our experiments. Concomitant evaluation of the miRNA expression levels by RT-qPCR showed a statistically significant increase of hsa-miR-1229-3p but not hsa-miR-1226-3p or hsa-miR-1227-3p in response to SRSF1 overexpression (Fig. 4b, the diagram at the left, and Table 4). Whereas a significant upregulation of SRSF2-dependent expression was observed for hsa-miR-1227-3p (*p* = 0.008) and miR-1229-3p (*p* = 0.008), the hsa-miR-1226-3p did not exhibit statistically different (*p* = 0.15) expression levels between the HCT116 line and the line overexpressing the SRSF2 SF (Fig. 4b, the diagram at the right). A bioinformatic analysis of the splicing signals using the Human Splicing Finder online tool [27] identified putative consensus motifs typical for SRSF1 and SRSF2 in exons proximal to hsa-miR1227- and hsa-miR1229-containing introns, respectively (Additional file 1: Figure S3). These findings are in agreement with the observation that the overexpression of SRSF1 had the largest impact on the expression of hsa-miR-1229-3p, whereas SRSF2 on hsa-miR-1227-3p. SRSF1-specific and SRSF2-specific enhancers established nearby hsa-miR-1226-3p may point to the presence of a complex regulatory element with overlapping enhancer and silencer functions causing uneven expression levels of this miRNA in different biological repeats and high *p* values.

**Table 4:** Change of mirtronic miRNA expression levels in response to the overexpression of splicing factors

Overexpressed splicing factor	Change of mirtronic miRNA expression levels, folds (*p* value)		
	miR-1226	miR-1227	miR-1229
SRSF1 (3.2 ± 0.6-fold)	1.5 ± 0.6 (*p* = 0.23)	−1.1 ± 0.4 (*p* = 0.47)	1.7 ± 0.4^a^ (*p* = 0.05)
SRSF2 (1.6 ± 0.5-fold)	1.5 ± 0.8 (*p* = 0.15)	1.9 ± 0.7^a^ (*p* = 0.008)	1.4 ± 0.3^a^ (*p* = 0.008)

A significance level of 0.05 was applied as a cutoff value

^a^Statistically significant results

Overall, our results lead to a conclusion that altered expression of a particular SF may affect the levels of mirtronic miRNAs in the cell.

## Discussion

To date, nearly 500 human miRNA-containing mirtrons have been predicted by bioinformatic analysis of deep sequencing data [14]. Nevertheless, their dependence on splicing was experimentally validated only for two human miRNAs located in conventional mirtrons, hsa-miR-877-5p and hsa-miR-1226-3p [16, 17]. Moreover, there is no convincing evidence for the participation of splicing machinery in biogenesis of miRNAs processed from 5′-tailed mirtrons, the most abundant subtype accounting for 86 % of all predicted mirtrons [14]. Likewise, for 3′-tailed mirtronic miRNAs, the splicing dependency was determined only in *Drosophila* [28]. Therefore, direct experimental evidence is necessary to confirm assignment of miRNAs to the mirtron pathway. In the present study, we experimentally proved that hsa-miR-1227-3p, hsa-miR-1229-3p, and hsa-miR-1236-3p derived from conventional mirtrons are processed by the splicing machinery. Meanwhile, splicing was not strictly required for the maturation of hsa-miR-1238-3p, previously annotated as mirtron-encoded miRNA [12, 15]. Indeed, Wen et al. recently reclassified the hsa-mir-1238 mirtron in miRBase based on more stringent bioinformatic criteria applied for mirtron evaluation [14]. Despite successful experimental validation of mirtronic miRNAs derived from conventional mirtrons, analysis of the annotated 5′-tailed mirtrons containing hsa-miR-3064-5p and hsa-miR-6515-5p or 3′-tailed mirtrons with hsa-miR-3940-5p and hsa-miR-6850-5p revealed no splicing requirement for the biogenesis of the intron-derived miRNAs. Thus, it is still an open question whether 5′- or 3′-tailed mirtrons actually exist in mammals. Overall, five out of nine tested human mirtrons generate miRNA irrespective of splicing. Similarly, several groups reported that only two of three [29] and two of four [16] predicted mirtronic miRNA candidates mature via a mirtron biogenesis pathway. These observations highlight the relevance of experimental validation of predicted mirtrons.

Mirtron-encoded miRNAs were predominantly found to occur in young, recently emerged genes [30]. Despite their relatively short evolution, the newly evolved miRNAs are incorporated into beneficial regulatory networks [31]. Babiarz et al. suggested that mirtronic miRNAs play important functional roles in post-mitotic neurons in the mammalian brain [32]; however, very little is known about their expression in cancerous cells. In this study, cell line-specific profiles of miRNAs processed from conventional mirtrons, hsa-miR-1226-3p, hsa-miR-1227-3p, hsa-miR-1229-3p, and hsa-miR-1236-3p, in seven various pancreatic, kidney, colorectal, and stomach cancer cell lines were identified (Table 2). The miRNA variability in cancer cell lines was determined by changes in abundance of two mirtronic miRNAs, hsa-miR-1229-3p and hsa-miR-1236-3p, while the expression levels of hsa-miR-1226-3p and hsa-miR-1227-3p declined in all tested cancer cell lines compared to those in HEK 293A. Remarkably, hsa-mir-1229-3p is upregulated in cell lines derived from metastatic sites and downregulated in primary tumor lines. However, further studies are required to assess whether miR-1229-3p is associated with the metastatic potential of human tumors.

To further increase the predictive value for clinical applications, we extended studies of these mirtronic miRNAs to a number of primary pancreatic, colorectal, stomach cancerous and healthy tissues (Fig. 3). We presume that analysis of non-paired cohorts of tumoral and non-tumoral samples allows a better estimation of miRNA expression changes, since healthy tissues are certainly not affected by cancerogenesis. Three mirtron-derived miRNAs showed organ-specific profiles in cancerous tissues of the digestive system. T3 stage pancreatic tumors exhibited a decline of the hsa-miR-1227-3p and hsa-miR-1236-3p expression. The levels of hsa-miR-1226-3p decreased in the T3 stage colorectal but conversely increased in the stomach cancer tissues. Remarkably, hsa-mir-1229-3p, which was upregulated in the metastatic site-derived cell lines, exhibited no expression change in the primary tumors. Previous studies demonstrated that cellular levels of hsa-miR-1226-3p and hsa-miR-1236-3p inversely correlate with cancerogenesis in breast and hepatoma cancer cells, respectively [20, 33]. Taken together, these results suggest that dysregulation of conventional mirtronic miRNAs could lead to the initiation and progression of specific human cancers. Moreover, the development of new medical applications can be based on artificial mirtron-mediated gene silencing platforms. As promising and affordable tools, they surpass conventional RNA interference (RNAi) approaches and allow to consolidate the high therapeutic potential of RNAi and protein-coding genes in specific cell types [34–36]. For example, hsa-miR-1226-3p processed from an artificial conventional mirtron effectively silences aberrant myotonic dystrophy protein kinase [35], mmu-miR-1224 leucine-rich repeat serine/threonine-protein kinase 2, and α-synuclein Parkinson disease-associated genes [36], while an artificial 3′-tailed mirtron knocks down the expression of the vascular endothelial growth factor A [34].

It is known from previous studies that canonical miRNA biogenesis and splicing mechanisms are directly coupled [37]. The binding of splicing regulatory proteins SRSF1 and hnRNPA1 to the stem loop of pri-miR-7 and pri-miR-18a, respectively, appears to affect Drosha processing of primary miRNA transcripts [26, 33]. Similarly, KHSPR promotes the maturation of a subset of miRNA precursors, such as pre-let-7a, pre-mir-1, and pre-mir-15 [38]. Consequently, SFs regulate both splicing and canonical miRNA processing, but little is known about their function in the biogenesis of miRNAs hosted on mirtrons. It has been only observed in patients with myelodysplastic syndrome that SF3B1 and SRFS2 mutations are associated with a downregulated expression of miRNAs derived from the putative 5′-tailed mirtrons hsa-miR-3605-5p and hsa-miR-4728-5p, respectively [5].

Our results revealed that increased nuclear levels of SRSF1 in tested HCT116 cells significantly enhanced the hsa-miR-1229-3p expression, while upregulated SRSF2 increased the abundance of the mirtronic miRNAs hsa-miR-1227-3p and hsa-miR-1229-3p. To our knowledge, these data, for the first time, demonstrate that an individual SF can directly act as a positive regulator for particular species of mirtronic miRNAs.

To gain insight into the regulation of mirtronic miRNA by components of splicing machinery, we correlated the mirtronic miRNA levels with the expression profiles of five SFs in different human digestive and excretory system cell lines or pancreatic, colorectal, and stomach tissues. It should be noted that RNA (this paper) and proteins [39] were purified simultaneously from the same tissue or cell line samples. The experiments revealed a reduced expression of SRSF1 and SRSF2 in pancreatic, kidney, colorectal, and stomach cancer cell lines as well as in cancer tissues — SRSF2 was downregulated in stomach tumors, while SRSF1 was reduced in all types of tested tumors [39]. Accordingly, tumor-promoting roles of SRSF1 and SRSF2 have been reported in various cancer types [40, 41]. Still, reduced expression of SRSF1 and SRSF2 proteins in the cancer cells [39] weakly correlated with signature of mirtronic miRNA expression (Tables 2 and 3). For example, no obvious differences in abundance of hsa-miR-1229-3p were seen among samples collected from the pancreas, colon, or stomach, notwithstanding a ~14-fold under-expression of SRSF1 and a sevenfold of SRSF2 in the colorectal and stomach cancerous tissues. At a first glance, these results seem to contradict the finding that these SFs positively modulate the levels of mirtronic miRNAs under overexpression conditions in the model cell line HCT116 (Fig. 4). However, first, a dual role for SRSF1 as a splicing activator and repressor was proposed [42]. Second, the expression of other factors involved in the regulation of splicing, U2AF35, U2AF65, and KHSRP, also significantly varied among cell lines and tumors [39]. In addition, the spliceosome mutations may affect the expression of miRNA genes [5]. Thus, biogenesis of mirtronic miRNAs could be regulated by different SFs or by a network of antagonistic and collaborative interactions of spliceosome components. In general, our data imply that a combination of multiple SFs may differently affect the biogenesis of mirtron-derived miRNAs, making it extremely difficult to predict particular effects of individual spliceosome components on the mirtron processing.

## Conclusions

In this study, we experimentally validated three novel splicing-dependent miRNAs processed from mirtrons, hsa-miR-1227-3p, hsa-miR-1229-3p, and hsa-miR-1236-3p. The expression analysis, for the first time, identified specific profiles of mirtronic miRNAs in cancerous cells and tumors suggesting their potential for exploiting as diagnostic tools. Finally, we demonstrated that the SRSF1 and SRSF2 SFs contribute to the biogenesis of mirtronic hsa-miR-1227-3p and hsa-miR-1229-3p.

## Methods

### Human cell cultures

HEK 293A (embryonic kidney), PANC-1 (pancreas/duct, epithelioid carcinoma), SU.86.86 (pancreas, ductal carcinoma; derived from a metastatic site: liver), T3M4 (pancreas, ductal carcinoma; metastasis), CaKi-1 (kidney, clear cell carcinoma, derived from a metastatic site: skin), 786-O (kidney, renal cell adenocarcinoma), HCT116 (colon, colorectal carcinoma), and KATOIII (stomach, gastric carcinoma; derived from a metastatic site: pleural effusion and supraclavicular and axillary lymph nodes and Douglas cul-de-sac) cells were cultured in the RPMI 1640 medium supplemented with 10 % fetal bovine serum and penicillin (100 U/ml) and streptomycin (100 μg/ml).

### Tumors and healthy tissues

Collection of surgically removed tumor and healthy tissue samples was described previously [39]. In total 29 healthy (8 pancreatic, 6 colorectal, 15 stomach) ant 37 tumor (12 pancreatic, 12 colorectal, 13 stomach) tissues were used for the experiments. The experiments were undertaken with the understanding and written consent of each subject, and the study conforms to The Code of Ethics of the World Medical Association (Declaration of Helsinki).

### Construction of plasmids

Minigene constructs of 1226/DHX30 (from exon 19 to exon 21; mirtronic intron 20), 1227/PLEKHJ1 (exons 5 to 7; mirtronic intron 5), 1229/MGAT4B (exons 11 to 13; mirtronic intron 12), 1236/NELFE (exons 3 to 4; mirtronic intron 3), 1238/ATG4D (exons 7 to 9; mirtronic intron 8), 3064/DDX5 (exons 10 to 12, mirtronic intron 11), 3940/KHSRP (exons 13 to 15, mirtronic intron 14), 6515/CARL (exons 5 to 7, mirtronic intron 5), and 6850/RPL8 (exons 2 to 4, mirtronic intron 2) were amplified from human Jurkat cell genomic DNA (ThermoFisher Scientific) using Phusion High-Fidelity DNA Polymerase (ThermoFisher Scientific) according to the manufacturer’s instructions. PCR products were digested with BamHI and HindIII restriction endonucleases, except 3940/KHSRP, 6515/CARL (digested with HindIII and EcoRI), and 6850/RLP8 (EcoRI and NotI), and inserted into pcDNA3 plasmid. PCR introduced 5′- and 3′-splice site mutations by using Phusion High-Fidelity DNA Polymerase with primers containing splicing site mutations (Additional file 2: Table S2). Amplification products were circularized using T4 DNA ligase (all enzymes were obtained from ThermoFisher Scientific). All final plasmid constructs were confirmed by Sanger sequencing.

The expression plasmids pSRSF1 and pSRSF2 were constructed by insertion of SRSF1 isoform 1 and SRSF2-coding DNA sequence into the pcDNA3 vector, respectively. Total RNA, extracted from HCT116 cells, was converted to complementary DNA (cDNA) using RevertAid RT kit (ThermoFisher Scientific). The CDS of genes were PCR-amplified using primers containing HindIII and BamHI sites (Additional file 2: Table S1), digested with appropriate restriction enzymes and the resulting fragments ligated into pcDNA3. Clones were confirmed by DNA sequence analysis.

### Transfection

HTC116 cells were cultured in RPMI 1640 media. Transient transfections were performed using Lipofectamine LTX transfection reagent (Invitrogen) according to the manufacturer’s instructions. For overexpression assay, HTC116 cells were seeded in 60-mm plates and allowed to grow for 24 h prior to transfection with pcDNA3-SRSF1, pcDNA3-SRSF2, pcDNA3-DHX30, pcDNA3-PLEKHJ1, pcDNA3-MGAT4B, pcDNA3-NELFE, pcDNA3-ATG4D, pcDNA3-DDX5, pcDNA3-KHSRP, pcDNA3-CARL, or pcDNA3-RPL8 constructs. A pcDNA3 vector was transfected to be used as a RT-qPCR reference. RNAs and proteins were isolated after 48 h. The experiments were independently repeated three times.

### RNA isolation

RNA from cell lines was isolated using an RNAzol^®^ RT reagent according to the manufacturer’s instructions (Molecular Research Center). Native proteins for SF studies by Jakubauskienė et al. [39] and total RNA used for the miRNA analysis (present paper) from tumors and healthy tissues were purified from the same sample using mirVana™ PARIS™ kit (ThermoFisher Scientific) according to the manufacturer’s instructions. Fraction of short RNAs (<200 nt) was used for miRNA RT and qPCR experiments. The integrity of long RNAs (>200 nt) was assessed using an Agilent 2100 Bioanalyzer or agarose (1 %) gel electrophoresis. To avoid DNA contamination before RT reaction, the RNA samples were treated with DNase I (ThermoFisher Scientific) according to the manufacturer’s instructions.

### Real-time RT-PCR of miRNAs

Each 20 μl of the RT reaction in the RT buffer contained 200 units of RevertAid Reverse Transcriptase (ThermoFisher Scientific), 1 mM dNTP (ThermoFisher Scientific), 1 μM RT primer mixture (Metabion), 16 units of RNase inhibitor RiboLock (ThermoFisher Scientific), and 200 ng of RNA. The mixture was incubated for 20 min at 25 °C and for 60 min at 37 °C and then heat-inactivated for 10 min at 70 °C. Real-time PCR was performed with SYBR Green PCR Master Mix (ThermoFisher Scientific) (35 cycles): initial denaturation for 10 min at 95 °C, followed by three cycles of amplification 15 s at 95 °C, 1 min at 55 °C, and 30 s at 60 °C, then 32 cycles 10 s at 95 °C and 30 s at 60 °C. Relative quantification of changes in the miRNA expression levels was performed using the comparative *C*_t_ (threshold cycle) method with normalization to the expression of endogenous control RNU48. Specific miRNA and RNU48 primers are provided in Additional file 2: Table S3. The RT primer mixture was composed of two primers: specific miRNA and RNU48. Quantitative PCR analysis was carried out on Rotor-Gene 6000 (Corbett Life Science) equipment. Part of RT-PCR products were inserted into pUC19 plasmid, and constructs were confirmed by sequence analysis.

### Detection of spliced and unspliced forms of mRNA

Spliced and unspliced DHX30, PLEKHJ1, MGAT4B, NELFE, ATG4D, DDX5, KHSRP, CARL, and RPL8 mRNA isoforms were detected by RT-PCR. cDNA synthesis was carried out using RevertAid Reverse Transcriptase and random hexamer primers according to the manufacturer’s (ThermoFisher Scientific) instructions. PCR was carried out using TrueStart Hot Start Taq DNA Polymerase (ThermoFisher Scientific) according to the manufacturer’s instructions. Amplification products were separated on 1 % agarose gel. TBP was used as loading control. Sequences of primers specific for the outer exons of mirtronic introns are provided in Additional file 2: Table S4.

### Protein preparation from cell cultures and western blotting

The nuclear fractions of proteins for western blotting were prepared from cell cultures using the NE-PER Nuclear and Cytoplasmic Extraction Reagents kit (ThermoFisher Scientific) according to the manufacturer’s instructions. Proteins were separated on a 10 % SDS–polyacrylamide gel and transferred onto the membrane. The membrane was incubated with appropriate primary antibody: anti-SRSF1 (Santa Cruz), anti-SRSF2 (Abcam), or anti-β-actin (Abcam), and washed and incubated with secondary antibody (Dako). The membrane was developed using TMB reagent (Sigma-Aldrich). All data were quantitated using MultiGauge software (Fujifilm). β-Actin expression was used for data normalization.

### Statistical analysis

Statistical analysis was performed using Sigma Plot software v. 11. Two-tailed unpaired Student’s *t* test and Mann-Whitney rank sum test were used to compare the differences in distribution between experimental results. A value of *p* < 0.05 was considered statistically significant.

## Additional files

Additional file 1: Figure S1. Splicing-dependent and Drosha-independent biogenesis of mirtronic miRNAs. The spliceosome, a multi-component ribonucleoprotein complex, cuts out the introns from pre-mRNA transcripts and splices together the exons resulting in the maturation of messenger RNA. Lariats of the introns are debranched by lariat debranching enzyme. The produced structures fold into pre-miRNA hairpins that are exported to the cytoplasm. After the cleavage by Dicer, the guide miRNA strand of mature miRNA/miRNA* duplex is loaded into functional Ago complex. **Figure S2.** Schematic representation of human genes containing mirtronic miRNAs. Schemes represent protein-coding genes, harboring reside-verified mirtronic miRNA hsa-miR-1226-3p, putative conventional mirtronic miRNAs hsa-miR-1227-3p, hsa-miR-1229-3p, and hsa-miR-1238-3p, mirtronic miRNAs, located in 5′-tailed mirtrons hsa-miR-3064-5p and hsa-miR-6515-5p and mirtronic miRNAs, located in 3′-tailed mirtrons hsa-miR-3940-5p and hsa-miR-6850-5p. Green boxes and lines indicate exons and introns of protein-coding genes, respectively. Purple boxes indicate 5′ and 3′ untranslated regions. Mirtrons containing previously mentioned mirtronic miRNAs are depicted as red lines. Full sequence of mirtrons (except of hsa-miR-3064) is written above each scheme, where sequence of more abundant miRNA of the hairpin is marked in red, less abundant in blue, and tail is marked in green. Dashes beneath the schemes show minigenes positions in the genes. **Figure S3.** Expression of mirtronic miRNAs in cancer cell lines. miRNA expression normalized to RNU48 and compared to HEK 293A. The experiments were performed in at least three biological replicates. The error bars represent calculated values for standard deviation. Fold changes higher than 1.5 were statistically significant (*p* < 0.05). **Figure S4.** The position of splicing signals predicted by online bioinformatics tool Human Splicing Finder. SRSF1-specific targets are highlighted in yellow, SRSF2-specific in green. Sequence of mirtrons is written as italicized red text, exon sequence normal black text. (PDF 750 kb) Additional file 2: Table S1. Primers for DHX30, PLEKHJ1, MGAT4B, NELFE, ATG4D, DDX5, KHSRP, CARL, and RPL8 gene amplification. **Table S2.** Primers used for 5′ and 3′ splicing site mutagenesis. **Table S3.** Specific miRNA and RNU48 primers for RT-qPCR. **Table S4.** Primers for spliced and unspliced gene form detection. (PDF 393 kb)
