# Supplementary material for: Splicing-dependent expression of microRNAs of mirtron origin in human digestive and excretory system cancer cells
Source: Clin Epigenetics. 2016 Mar 25;8:33. doi: 10.1186/s13148-016-0200-y (PMC4807562; doi:10.1186/s13148-016-0200-y)
Supplement: Additional file 1: Figure S1. — Splicing-dependent and Drosha-independent biogenesis of mirtronic miRNAs. The spliceosome, a multi-component ribonucleoprotein complex, cuts out the introns from pre-mRNA transcripts and splices together the exons resulting in the maturation of messenger RNA. Lariats of the introns are debranched by lariat debranching enzyme. The produced structures fold into pre-miRNA hairpins that are exported to the cytoplasm. After the cleavage by Dicer, the guide miRNA strand of mature miRNA/miRNA* duplex is loaded into functional Ago complex. Figure S2. Schematic representation of human genes containing mirtronic miRNAs. Schemes represent protein-coding genes, harboring reside-verified mirtronic miRNA hsa-miR-1226-3p, putative conventional mirtronic miRNAs hsa-miR-1227-3p, hsa-miR-1229-3p, and hsa-miR-1238-3p, mirtronic miRNAs, located in 5′-tailed mirtrons hsa-miR-3064-5p and hsa-miR-6515-5p and mirtronic miRNAs, located in 3′-tailed mirtrons hsa-miR-3940-5p and hsa-miR-6850-5p. Green boxes and lines indicate exons and introns of protein-coding genes, respectively. Purple boxes indicate 5′ and 3′ untranslated regions. Mirtrons containing previously mentioned mirtronic miRNAs are depicted as red lines. Full sequence of mirtrons (except of hsa-miR-3064) is written above each scheme, where sequence of more abundant miRNA of the hairpin is marked in red, less abundant in blue, and tail is marked in green. Dashes beneath the schemes show minigenes positions in the genes. Figure S3. Expression of mirtronic miRNAs in cancer cell lines. miRNA expression normalized to RNU48 and compared to HEK 293A. The experiments were performed in at least three biological replicates. The error bars represent calculated values for standard deviation. Fold changes higher than 1.5 were statistically significant (p < 0.05). Figure S4. The position of splicing signals predicted by online bioinformatics tool Human Splicing Finder. SRSF1-specific targets are highlighted in yellow, SRSF2-specific in green. [file 13148_2016_200_MOESM1_ESM.pdf]

ADDITIONAL FILE 1

Splicing-dependent expression of microRNAs of mirtron origin in human digestive and excretory system cancer cells

Stasė Butkytė[1], Laurynas Čiupas[1], Eglė Jakubauskienė[2], Laurynas Vilys[2], Paulius Mocevicius[3], Arvydas Kanopka[2] and Giedrius Vilkaitis[1*]

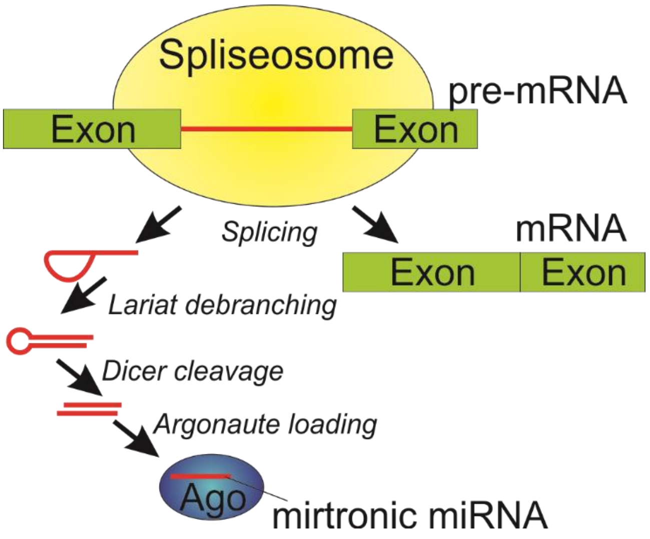

Figure S1. Splicing-dependent and Drosha-independent biogenesis of mirtronic miRNAs. The spliceosome, a multi-component ribonucleoprotein complex, cuts out the introns from pre-mRNA transcripts and splices together the exons resulting in the maturation of messenger RNA. Lariats of the introns are debranched by lariat debranching enzyme. The produced structures fold into pre-miRNA hairpins that are exported to the cytoplasm. After the cleavage by Dicer the guide miRNA strand of mature miRNA/miRNA* duplex is loaded into functional Ago complex.

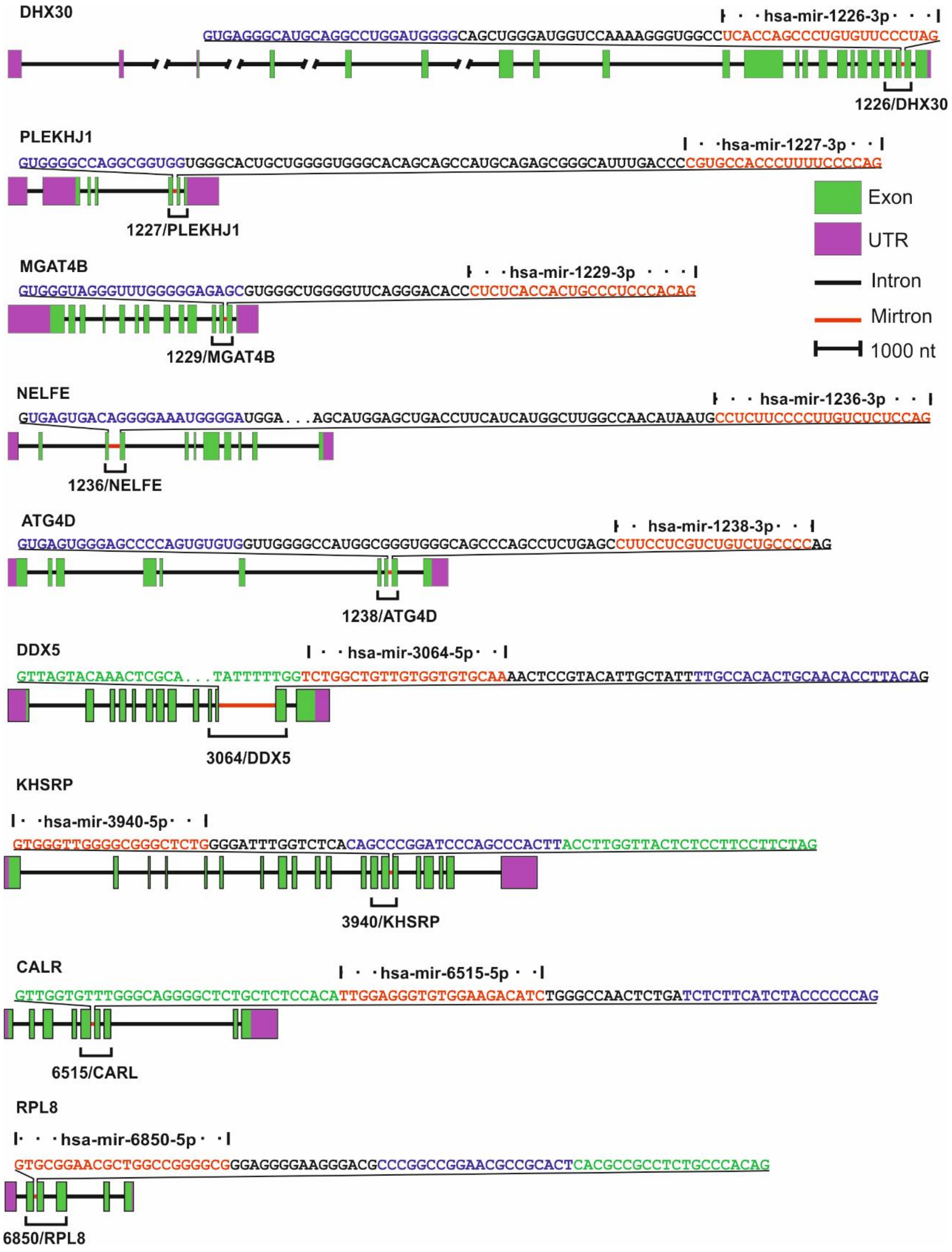

Figure S2. Schematic representation of human genes containing proven and putative mirtronic miRNAs. Schemes represent protein-coding genes, harboring verified mirtronic miRNA hsa-miR-1226-3p, putative conventional mirtronic miRNAs hsa-miR-1227-3p, hsa-miR-1229-3p, hsa-miR-1238-3p, mirtronic miRNAs, located in 5'-tailed mirtrons hsa-miR-3064-5p, hsa-miR-6515-5p and mirtronic miRNAs, located in 3'-tailed mirtrons hsa-miR-3940-5p, hsa-miR-6850-5p. Green boxes and lines indicate exons and introns of protein-coding genes, respectively. Purple boxes indicate 5' and 3' untranslated regions. Mirtrons containing previously mentioned mirtronic miRNAs are depicted as red lines. Full sequence of mirtrons (except of hsa-miR-3064) is written above each scheme, where sequence of more abundant miRNA of the hairpin is marked in red, less abundant in blue and tail is marked in green. Dashes beneath the schemes show minigenes positions in the genes.

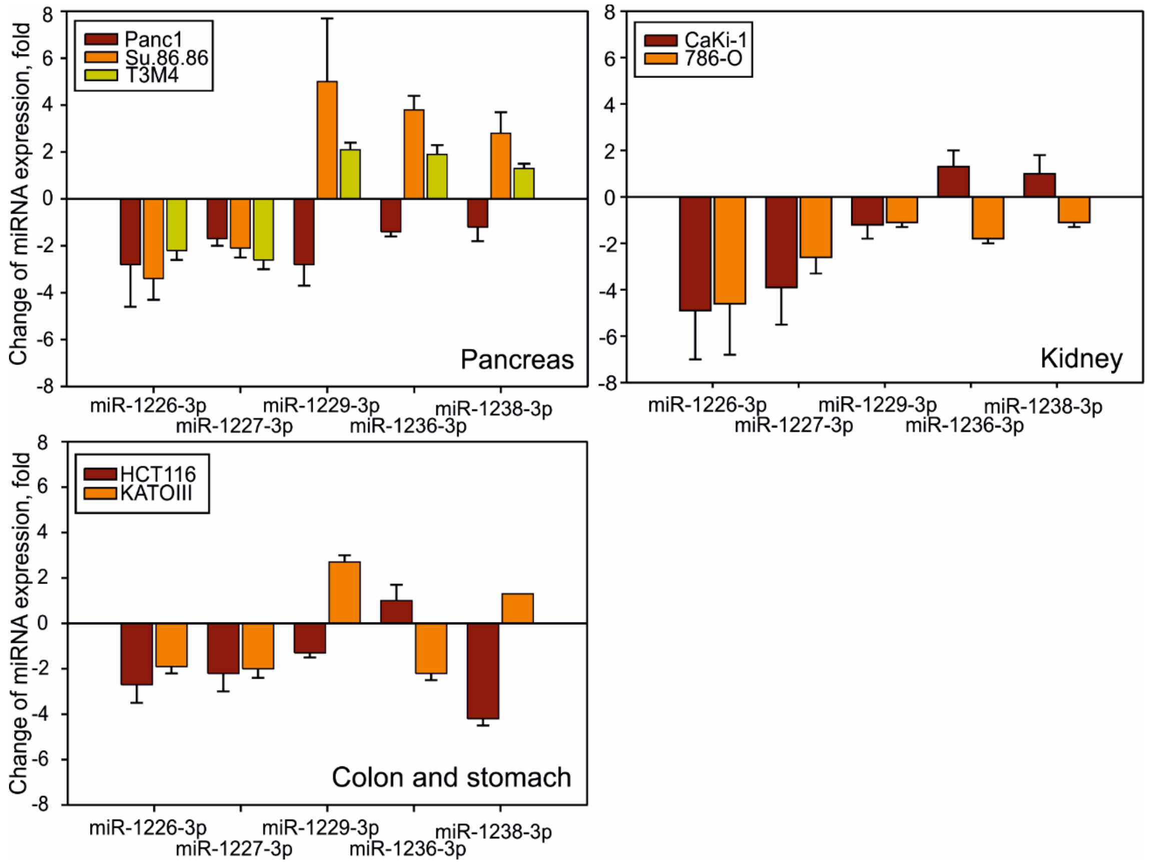

Figure S3. Expression of mirtronic miRNAs in cancer cell lines. miRNAs expression normalized to RNU48 and compared to HEK 293A. At least 3 biological replicates were used for experiments. Error bars represent calculated values for standard deviation. Fold changes higher than 1.5 were statistically significant ($p<0.05$).

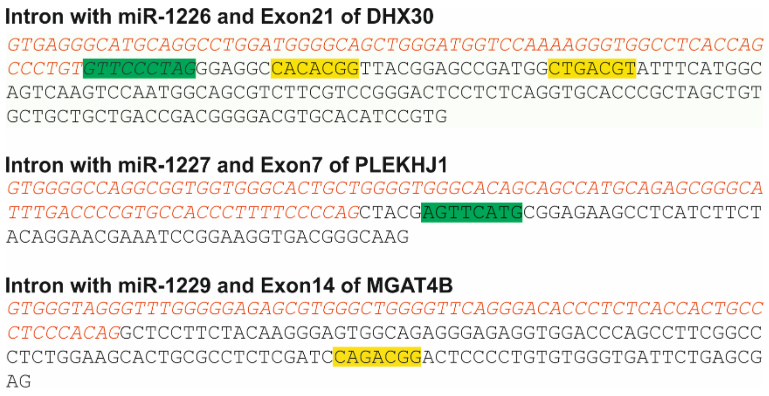

Figure S4. The position of splicing signals predicted by online bioinformatics tool Human Splicing Finder. SRSF1-specific targets are highlighted in yellow, SRSF2-specific – in green. Sequences of mirtrons are written as italicized red text, exon sequences – normal black text.
