# Supplementary material for: Splicing-dependent expression of microRNAs of mirtron origin in human digestive and excretory system cancer cells
Source: Clin Epigenetics. 2016 Mar 25;8:33. doi: 10.1186/s13148-016-0200-y (PMC4807562; doi:10.1186/s13148-016-0200-y)
Supplement: Additional file 2: Table S1. — Primers for DHX30, PLEKHJ1, MGAT4B, NELFE, ATG4D, DDX5, KHSRP, CARL, and RPL8 gene amplification. Table S2. Primers used for 5′ and 3′ splicing site mutagenesis. Table S3. Specific miRNA and RNU48 primers for RT-qPCR. Table S4. Primers for spliced and unspliced gene form detection. (PDF 393 kb) [file 13148_2016_200_MOESM2_ESM.pdf]

ADDITIONAL FILE 2

Splicing-dependent expression of microRNAs of mirtron origin in human digestive and excretory system cancer cells

Stasė Butkytė[1], Laurynas Čiupas[1], Eglė Jakubauskienė[2], Laurynas Vilys[2], Paulius Mocevicius[3], Arvydas Kanopka[2] and Giedrius Vilkaitis[1*]

Table S1. Primers for DHX30, PLEKHJ1, MGAT4B, NELFE, ATG4D, DDX5, KHSRP, CARL, RPL8 genes amplification.

Gene	Primers
DHX30	FW 5'-CCCTAGAAGCTTACTCATCAAGCAGTTCTC-3'<br>REV 5'-ACCCACGGATCCCACGGATGTGCACGTCCC-3'
PLEKHJ1	FW 5'-CCCCAGAAGCTTGCTTCATTGAGGACCCTGAGAGG-3'<br>REV 5'-CTGCGCGGATCCTCACGCCTGCAAGCCACTC-3'
MGAT4B	FW 5'-CTGCAGCGAAGCTTGTTCTTCTTCCGCAGTGGGAAC-3'<br>REV 5'-CCTCACCGGGATCCCTCGCTCAGAATCACCCACACA-3'
NELFE	FW 5`- TCCCAGAAGCTTAAAAAGGCATTGCTGGCT-3`<br>REV 5`-GCTCACGGATCCCTTTAACTTCCCCTCAAG-3`
ATG4D	FW 5'-CTGCAGAAGCTTGAACTCCTGCGTTGCGAGCT-3'<br>REV 5'-GCTCACGGATCCCCTGGTCAGCTCTGAGCAGAGTG-3'
DDX5	FW 5‘-ACTTAGAAGCTTGTGGCCTGCCATGGGTAT-3‘<br>REV 5‘-CCTTACGGATCCCTGAACCTCTGTCTTCGA-3‘
KHSRP	FW 5‘-TTGCAGAAGCTTAGTGGTCCCCCAGGTCCT-3‘<br>REV 5‘-ACTCACGAATTCTGTGGGGGAGCCCCGGGT-3‘
CARL	FW 5‘-CTTCAGAAGCTTGATGATGAGTTTACACACCT-3‘<br>REV 5‘-TCTCACGAATTCCTGCCAGAGGTCCA-3‘
RPL8	FW 5‘-CCCCAGGAATTCGACCCGTCGCCATG-3‘<br>REV 5‘-ACTCACGCGGCCGCCAACCACAGCTCTGTTGGCTG-3‘
SRSF1	FW 5‘-AATTAAGCTTATGTCGGGAGGTGGTGTGATT-3‘<br>REV 5‘-AATTGGATCCTTATGTACGAGAGCGAGA-3‘
SRSF2	FW 5‘-AATTAAGCTTATGAGCTACGGCCGCCCC-3‘<br>REV 5‘-AATTGGATCCTTAAGAGGACACCGCTCCTTCC-3‘

Table S2. Primers used for 5' and 3' splicing sites mutagenesis.

**Gene**	**5'splice site mutation**
DHX30	FW 5‘-ACCATTAACAGCTGAGGGCATGCA-3<br>REV 5'-CGACTTGTGCAGCAGGATGTTGCC-3'
PLEKHJ1	FW 5‘-GCGTCGGGCCAGCTGGGGCCAGGCG-3‘<br>REV 5'-AGAGCCTCCATCCACTCCTGACAC-3'
MGAT4B	FW 5‘-CCTCCAGATCGCTGGGTAGGGTTT-3‘<br>REV 5'-TAGCCGTCGGGGCTCCGAGGGTAC-3'
NELFE	FW 5`-TGTCAAACGCTCTGAGTGACAGGG-3<br>REV 5`-CCACCTTGGCTGGTTGTGCTGCTG-3`
ATG4D	FW 5‘-TCCCCCTGGAGCTGAGTGGGAGCC-3‘<br>REV 5'-AGTCGGCCTGGCTGACATCCACAG-3'
DDX5	FW 5‘-CAGAGGGCTAGCTTAGTACAAACT-3'<br>REV 5‘-GAGGCCACATCTGTAGCAATC-3'
KHSRP	FW 5‘-AAAAGATCGAGCTGGGTTGGGGCG-3'<br>REV 5‘-CCTCGATAAGCTGCTTGGCGTGGT-3'
CARL	FW 5‘-CCAAGCCTGAGCTTGGTGTTTGGG-3'<br>REV 5‘-AGTCTGTGGGATCATCGATCTTGGCC-3'
RPL8	FW 5‘-GCATCGTCAAGCTGCGGAACGCT-3'<br>REV 5‘-CCTTGATGTAGCCGTGCCGCTCAG-3'
	**3'splice site mutation**
DHX30	FW 5‘-CTGTGTTCCCTACGGAGGCCACAC-3‘<br>REV 5'-GGCTGGTGAGGCCACCCTTTTGGA-3'
PLEKHJ1	FW 5'-TTCCCCACCTACGAGTTCATGCGG-3'<br>REV 5'-AAGGGTGGCACGGGGTCAAATGCC-3'
MGAT4B	FW 5‘-GCCCTCCCACACGCTCCTTCTACA-3‘<br>REV 5'-AGTGGTGAGAGGGTGTCCCTGAAC-3'
NELFE	FW 5`-TTGTCTCTCCACCACTATCAGAGC-3`<br>REV 5`-GGGGAAGAGGCATTATGTTGGCCA-3
ATG4D	FW 5‘-TGTCTGCCCCACTCCTTCCACTGC-3‘<br>REV 5'-GACGAGGAAGGCTCAGAGGCTGGG-3'
DDX5	FW 5‘-AACACCTTACACATGTGGAAGATG-3'<br>REV 5‘-GCAGTGTGGCAAAATAGCAATGTA-3'
KHSRP	FW 5‘-CCTTCCTTCTACGGTCCTCTCTGC-3'<br>REV 5‘-AGAGTAACCAAGGTAAGTGGGCTG-3'
CARL	FW 5‘-TCTACCCCCCACGACTGGGACAAG-3'<br>REV 5‘-TGAAGAGATCAGAGTTGGCCCAGA-3'
RPL8	FW 5‘-CTCTGCCCACACGACATCATCCACGAC-3'<br>REV 5‘-GCGGCGTGAGTGCGGCGTT-3'

Table S3. Specific miRNA and RNU48 primers for RT-qPCR

miRNA	RT primer
hsa-miR-1226	5'-GTCGTATCCAGTGCAGGGTCCGAGGTATTCGCACTGGATACGACCTAGGG-3'
hsa-miR-1227	5'-GTCGTATCCAGTGCAGGGTCCGAGGTATTCGCACTGGATACGACCTGGGG-3'
hsa-miR-1229	5'-GTCGTATCCAGTGCAGGGTCCGAGGTATTCGCACTGGATACGACCTGTGG-3'
hsa-miR-1236	5'-GTCGTATCCAGTGCAGGGTCCGAGGTATTCGCACTGGATACGACCTGGAG-3'
hsa-miR-1238	5'-GTCGTATCCAGTGCAGGGTCCGAGGTATTCGCACTGGATACGACGGGGCA-3'
hsa-miR-3064	5'-GTCGTATCCAGTGCAGGGTCCGAGGTATTCGCACTGGATACGACTTGCAC-3'
hsa-miR-3940	5'-GTCGTATCCAGTGCAGGGTCCGAGGTATTCGCACTGGATACGACCAGAGC-3'
hsa-miR-6515	5'-GTCGTATCCAGTGCAGGGTCCGAGGTATTCGCACTGGATACGACGATGTC-3'
hsa-miR-6850	5'-GTCGTATCCAGTGCAGGGTCCGAGGTATTCGCACTGGATACGACCGCCCC-3'
RNU48	5'-GTCGTATCCAGTGCAGGGTCCGAGGTATTCGCACTGGATACGACGGTCAGAGC-3'

miRNA	Forward primer
hsa-miR-1226	5'-GCGTCACCAGCCCTGTGT-3'
hsa-miR-1227	5'-GCGCGTGCCACCCTTTT-3'
hsa-miR-1229	5'-GCGCTCTCACCACTGCCCTC-3'
hsa-miR-1236	5'-GCGCCTCTTCCCCTTGTCT-3'
hsa-miR-1238	5'-GCGCTTCCTCGTCTGTC-3'
hsa-miR-3064	5'-GCGTCTGGCTGTTGTGGT-3'
hsa-miR-3940	5'-GCGGTGGGTTGGGGCGG-3'
hsa-miR-6515	5'-GCGTTGGAGGGTGTGGAA-3'
hsa-miR-6850	5'-GTGCGGAACGCTGGCC-3'
RNU48	5'-GCGTGCCATCACCGCAGC-3'
	**Reverse primer**
	5'-GTGCAGGGTCCGAGGT-3'

Table S4. Primers for spliced and unspliced gene form detection.

Gene	Forward primer
DHX30	FW 5'-GTGAGGCAGGGCAAGGTCAC-3'<br>REV 5'-CACGGATGTGCACGTCCCCG-3'
PLEKHJ1	FW 5'-GCTTCATTGAGGACCCTGAG-3'<br>REV 5'-CTTGCCCGTCACCTTCCGGA-3'
MGAT4B	FW 5'- AACCCTCAGTCAGACAAGGA-3'<br>REV 5'-CTCGCTCAGAATCACCCACA-3'
NELFE	FW 5'-AGCTTAAAAAGGCATTGCTG-3'<br>REV 5'-GATCCCTTTAACTTCCCCTC-3'
ATG4D	FW 5'-ATGACTTCCTGCTGTACCTG-3'<br>REV 5'-CCTGGTCAGCTCTGAGCAGA-3'
DDX5	FW 5'-AATTCAAACATGGAAAAGCTCC-3'<br>REV 5'-CTGAACCTCTGTCTTCGACC-3'
KHSRP	FW 5'-GTGGCGAGAATGTGAAAGC-3'<br>REV 5'-CTGGTTGAAGGGCCCAGG-3'
CARL	FW 5'-CCTGTACACACTGATTGTGCG -3'<br>REV 5'-CTTGTACTCAGGGTTCTGAATCAC -3'
RPL8	FW 5'-CCATGGGCCGTGTGATCCG -3'<br>REV 5'-CCCGTGTGAATGCCCTCG -3'
TBP	FW 5'-TTCGGAGAGTTCTGGGATTGTA-3'<br>REV 5'-TGGACTGTTCTTCACTCTTGGC-3'
